# Plasma Hsp90 Level as a Marker of Early Acute Lymphoblastic Leukemia Engraftment and Progression in Mice

**DOI:** 10.1371/journal.pone.0129298

**Published:** 2015-06-11

**Authors:** Mateus Milani, Angelo Brunelli Albertoni Laranjeira, Jaíra Ferreira de Vasconcellos, Silvia Regina Brandalise, Alexandre Eduardo Nowill, José Andrés Yunes

**Affiliations:** 1 Laboratório de Biologia Molecular, Centro Infantil Boldrini, Campinas, SP, Brazil; 2 Centro Integrado de Pesquisas Oncohematologicas da Infância (CIPOI), Faculdade de Ciências Médicas (FCM), Universidade Estadual de Campinas (UNICAMP), Campinas, SP, Brazil; 3 Departamento de Genética Médica, FCM, UNICAMP, Campinas, SP, Brazil; European Institute of Oncology, ITALY

## Abstract

Current monitoring of acute lymphoblastic leukemia (ALL) in living mice is based on FACS analysis of blood hCD45+ cells. In this work, we evaluated the use of human IGFBP2, B2M or Hsp90 as soluble markers of leukemia. ELISA for B2M and IGFBP2 resulted in high background levels in healthy animals, precluding its use. Conversely, plasma levels of Hsp90 showed low background and linear correlation to FACS results. In another experiment, we compared Hsp90 levels with percentage of hCD45+ cells in blood, bone marrow, liver and spleen of animals weekly sacrificed. Hsp90 levels proved to be a superior method for the earlier detection of ALL engraftment and correlated linearly to ALL burden and progression in all compartments, even at minimal residual disease levels. Importantly, the Hsp90/hCD45+ ratio was not altered when animals were treated with dexamethasone or a PI3K inhibitor, indicating that chemotherapy does not directly interfere with leukemia production of Hsp90. In conclusion, plasma Hsp90 was validated as a soluble biomarker of ALL, useful for earlier detection of leukemia engraftment, monitoring leukemia kinetics at residual disease levels, and pre-clinical or mouse avatar evaluations of anti-leukemic drugs.

## Introduction

Patient-derived tumor xenografts mouse models has been largely used for the study of cancer biology, pre-clinical test of new drugs or new drug combinations, and more recently as avatars to pursue personalized therapeutic regimens [1]. Xenografts are usually obtained by subcutaneous implantation of small pieces of tumors into the flank of mice. In case of leukemia, xenografts are obtained by injection of 10 million cells into the tail vein or intrafemorally [2–5]. Subcutaneous tumor growth and drug response is easily monitored by measuring tumor volume with an external caliper, though with lower accuracy than more sophisticated imaging methods [6]. Monitoring leukemia xenografts is usually done by flow cytometry analysis of human CD45+ cells in peripheral blood [2–5]. However, leukemia homing and progression in nonobese diabetic (NOD)/SCID mouse occurs primarily in the bone marrow, liver and spleen [7]. Migration of leukemia cells into circulation is an active process controlled by SDF1/CXCR4 axis [8]. Consequently, the number of leukemia cells in peripheral blood may not always represent total leukemia burden, especially at earlier stages of leukemia engraftment and progression. Alternatively, high sensitivity methods for *in vivo* leukemia monitoring by bioluminescent or fluorescent imaging analysis require genetic modification of leukemia cells, which is not a straightforward method when handling with primary leukemia cells [9–12].

Soluble proteins secreted or released by leukemia cells into the circulation could be useful markers for earlier engraftment detection and to monitoring the dynamic growth of leukemia in mice. Serum levels of prostate-specific antigen (PSA) have been shown to correlate with tumor volume in animal models of prostate cancer [13]. Similarly, human specific lactate dehydrogenase (LDH) isoenzymes and the nuclear matrix protein 41/7 (NPM) were found to be useful serologic markers to monitor the dynamic growth of human leukemia in mice, though with low sensitivity in early stages of tumor growth. Detection of human pre-B acute lymphoblastic leukemia (ALL) cell line Nalm-6 was only possible when the number of Nalm-6 cells in bone marrow was equal or higher than 7.2% and 13.7%, respectively, of total cell numbers [14]. In this manuscript, we report on a highly sensitive method for detecting and monitoring ALL in mice by measuring plasma levels of human Hsp90.

## Materials and Methods

### Ethics statement

The institutional ethics committees approved this study for both humans and animals. The use of human samples in this study was approved by the human Research Ethics Committee from the State University of Campinas (CAAE 0014.0.144.146–08). Written informed consent could not be obtained due to death or lost follow-up. Animal use was approved by the Ethics Commission for Animal Use from Institute of Biology at State University of Campinas (CEUA/UNICAMP, protocol 2365–1).

### ALL cell samples

Experiments with primary ALL samples were performed with cryopreserved post-ficoll bone marrow mononuclear cells obtained from patients with newly diagnosed disease enrolled between 1991 to 2002.

RS4;11 and TALL-1 cells were cultured in RPMI-1640 medium, 10% fetal bovine serum (FBS), 20 IU/mL penicillin and 20 μg/mL streptomycin at 37°C and 5% CO2. Leukemia cell lines TALL-1 and RS4;11 were kindly provided by Dr. João Barata (Molecular Medicine Institute, Portugal) and Sheila A Shurtleff (St Jude Children’s Research Hospital, USA), respectively.

### Transplantation of NOD/SCID mouse with ALL cells

Primary ALL cells were thawed, washed with PBS and 1x10^7^ cells were injected via the tail vein in unconditioned NOD/SCID (NOD.CB17-Prkdcscid/J) mice (The Jackson Laboratory, Bar Harbor, ME) for an *in vivo* expansion step. Successfully engrafted mice were sacrificed, ALL cells were collected from spleen, liver and bone marrow and 1x10^7^ fresh cells were immediately injected in a higher number of secondary recipient mice for the experiments. In case of ALL cell lines, mice for the experiments were injected with cells expanded in vitro.

### Flow cytometry analysis of ALL engraftment and progression in NOD/SCID mice

After transplantation, animals were monitored every 7 days for ALL engraftment and progression. Blood was collected by retro-orbital bleeding into EDTA containing tube. Plasma was separated for ELISA and mononuclear cells were isolated by density gradient centrifugation using ficoll. ALL cells were detected by flow cytometry [7] in a FACSCanto II equipment (Becton Dickinson, Franklin Lakes, NJ), using anti-hCD45-PE (clone HI30, BD Pharmingen, San Diego, CA or EXBIO, Prague, Czech Republic) and anti-mCD45-FITC (clone 30F-11, BD Pharmingen).

For evaluation of ALL engraftment into organs, animals were sacrificed in an isoflurane chamber. Peripheral blood was immediately collected by cutting the renal vein. Bone marrow cells were obtained by flushing the femoral bones with PBS. Liver and spleen (whole organs) were mechanically homogenized and suspended with PBS. Post-ficoll mononuclear cells were analyzed as above. Total numbers of cells obtained were analyzed by flow cytometry.

### Enzyme-linked immunosorbent assay (ELISA)

Sandwich ELISA assays were performed in 96-well plates, using 100 μL peripheral blood plasma per well. When required, plasma was diluted in blocking buffer, in which case the concentration read from the standard curve was multiplied by the dilution factor. The following ELISA assays were used according to manufacturer recommendations: Hsp90α (human) ELISA Kit (Enzo Life Sciences, Farmingdale, NY), Human beta 2-Microglobulin Quantikine IVD ELISA Kit (R&D Systems Inc., Minneapolis, MN) and Human IGFBP-2 DuoSet (R&D Systems Inc.).

### Chemotherapy effect on Hsp90 levels

For chemotherapy interference experiment, mice were transplanted with a primary ALL cells and weekly monitored for ALL by flow cytometry and or ELISA Hsp90. In one experiment, animals were randomly distributed into different treatment groups (at least 3 animals per group), which received 30 mg/Kg of the PI3K inhibitor AS60524 [15], 5 mg/Kg of dexamethasone or vehicle (saline), intraperitoneally, once a day, 5 days a week. The water-soluble potassium salt of AS605240 was synthesized in the Chemistry Dept. at Federal University of Santa Catarina, according to patent WO 2004007491. Identity and purity were confirmed by mass spectrometry and nuclear magnetic resonance. Treatment started when human CD45+ cells reached ≥0.5% of peripheral blood cells in each of the animals. The number of human CD45+ cells in peripheral blood mononuclear cells and plasma levels of the ELISA biomarker (B2M, Hsp90 or IGFBP2) was measured at different time points, before and/or during treatment. Drug treatment was administered from 9 to 10 am, while blood sampling was done from 3 to 4 pm.

### Statistical Analysis

Plasma biomarker concentrations and percentage of ALL cells were transformed to log10 to behave as a normal distribution and tested for normality by the Kolmogorov-Smirnov and Shapiro-Wilk tests in SPSS 20.0 software. Associations of biomarker levels with percentage of ALL cells were evaluated by Mann Whitney test and/or Pearson correlation using GraphPad Prism 6 software. In some cases, percentage of ALL cells was considered using previously defined discrete categories.

## Results and Discussion

### Plasma Hsp90 stands out as an ALL biomarker in NOD/SCID mouse

**Fig 1:**
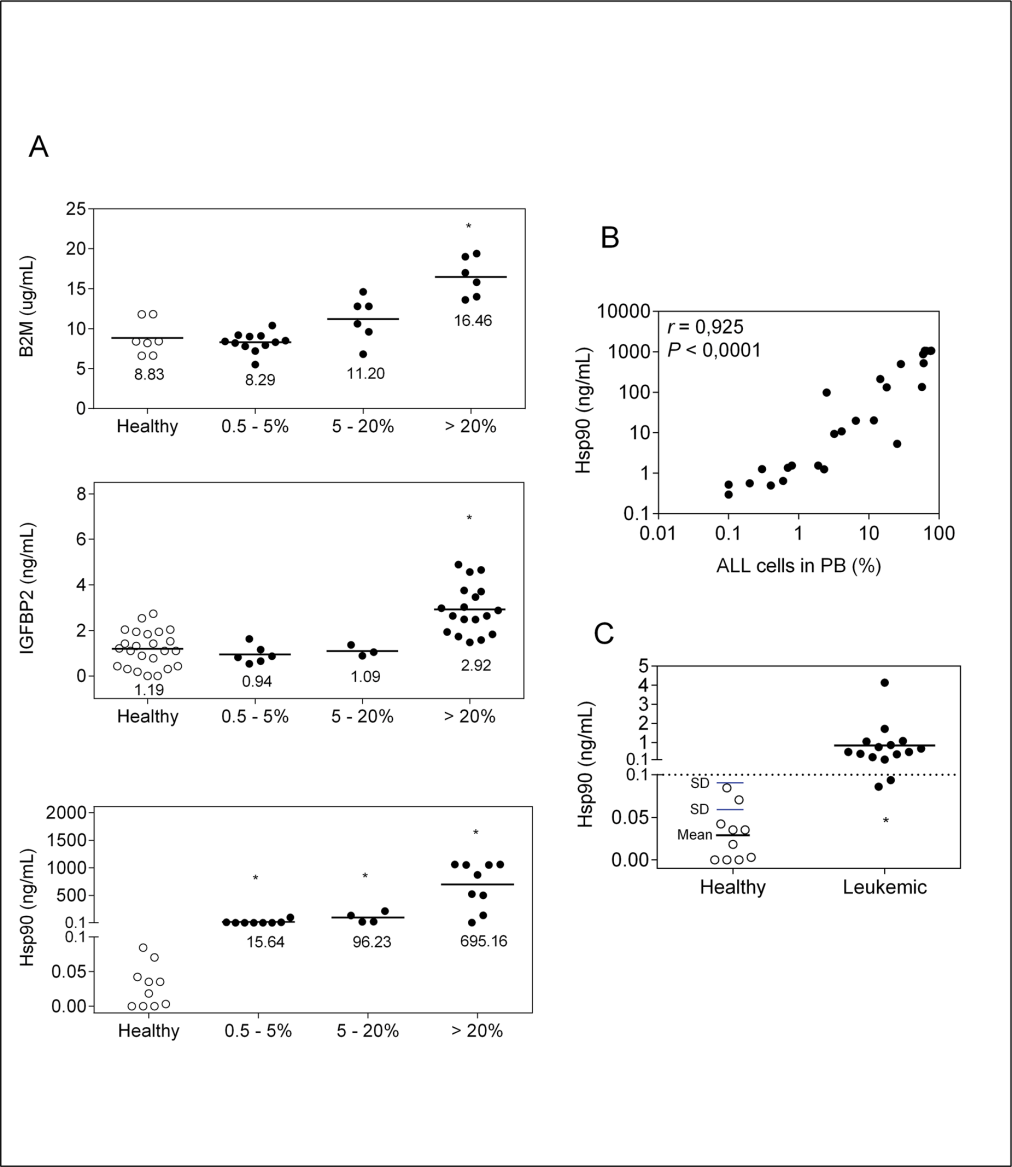
Analysis of three potential leukemia plasma biomarkers. (A) Peripheral blood B2M, IGFBP-2 and Hsp90 levels (ELISA) in NOD/SCID mice transplanted with a primary human T-ALL or healthy controls. Animals were divided into groups according to the percentage of ALL cells (hCD45+) in peripheral blood by FACS. Data points correspond to individual samples, and horizontal bars correspond to median. *P<0.05; Mann-Whitney U test. (B) Correlation between Hsp90 levels and % ALL cells in peripheral blood. Data points correspond to individual samples. Data were transformed to log10 and analyzed by Pearson’s correlation. (C) Cut-off value of Hsp90 (dotted line) as determined by adding 2 times SD to mean. Animals transplanted with different BCP-ALL (n = 3) and T-ALL (n = 2), sacrificed at the earliest time point (time point 1, Fig 2) were included in the analysis. Leukemia engraftment was confirmed by FACS analysis. Data points correspond to individual samples. PB, peripheral blood; SD, standard deviation.

Beta-2-microglobulin (B2M), insulin-like growth factor binding protein 2 (IGFBP2) and heat shock protein 90 (Hsp90) were selected as candidate soluble biomarkers of ALL based on their very high level of expression and extracellular secretion or release by leukemia cells [16–23] and also, for the availability of commercial ELISA kits. Analysis of these biomarkers levels quantified by ELISA, in plasma of healthy NOD/SCID mice *versus* mice transplanted with primary T-cell ALL (T-ALL), showed unacceptably high levels of B2M and IGFBP2 in healthy animals, suggesting antibodies cross-reactivity with the human and mouse biomarker. Even so, animals with high leukemia burden, i.e. high percentage of ALL cells in peripheral blood, had significantly higher levels of B2M and IGFBP2 (Fig 1A), indicating that these proteins are secreted/released at high levels by ALL cells in this mouse model. Importantly, plasma human B2M was present at micrograms concentration range, highlighting B2M as an attractive candidate for further studies provided that antibodies with higher specificity for the human protein are found.

Human Hsp90 levels were clearly higher in animals injected with ALL in comparison to healthy controls, even at the lowest percentage of human leukemia cells in the mice peripheral blood, which in this experiment was 0.5% (Fig 1A). Concentration of plasma Hsp90 levels reached microgram per milliliter amounts and a dynamic range of 4 logs. Moreover, there was a linear relationship between levels of human Hsp90 and percentage of peripheral blood ALL cells (Fig 1B). Based on background Hsp90 levels in healthy animals, a value of 0.1 ng/mL, which corresponds to the upper limit of the 95%CI (i.e. mean value added of 2 times the standard deviation) (Fig 1C), was taken as cut-off value for further experiments.

It is worth mentioning that the levels of human Hsp90 in bone marrow plasma samples from children in remission (end of therapy) were as high as in children at ALL diagnosis (data not shown). Therefore, in contrast to the mouse model, the background abundance of human Hsp90 precludes its use for ALL monitoring in patients.

### Plasma Hsp90 levels are representative of mice leukemic burden

The percentage of ALL cells in peripheral blood may not necessarily reflect bone marrow and secondary organ infiltration by leukemia. Usually, the beginning of exponential increase of ALL cells in peripheral blood corresponds to the end of log phase or even plateau phase in bone marrow [3,24].

**Fig 2:**
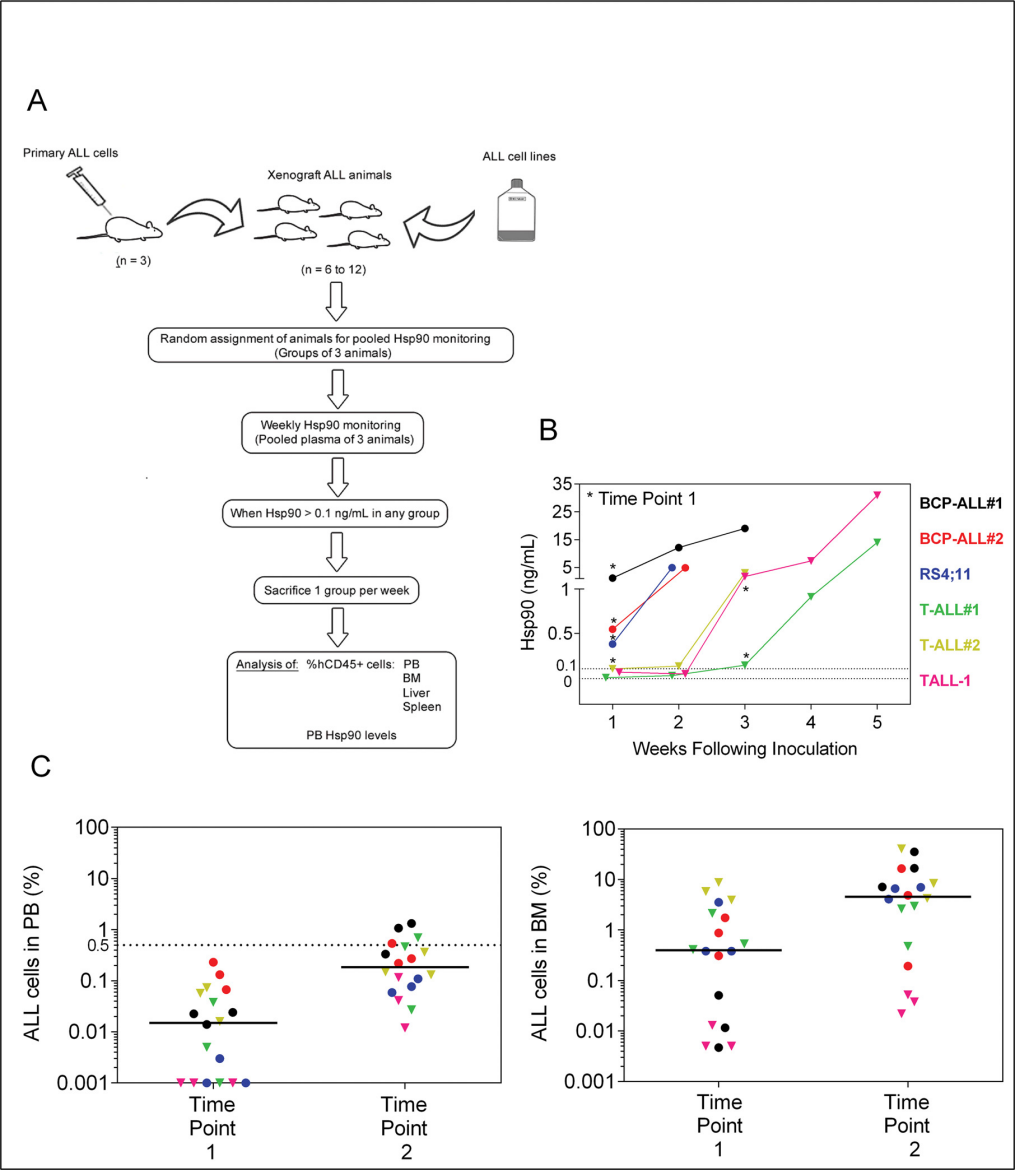
ELISA of plasma Hsp90 levels for earlier engraftment confirmation. (A) Experimental workflow. Cryopreserved primary ALL cells were thawed and injected into 3 mice for expansion. Fresh xenograph ALL cells were then injected into 6 to 12 secondary animals for experiments. For RS4;11 and TALL-1, cultured cells were directly injected in animals for the experiments. Animals were monitored weekly and sacrificed as indicated. (B) Kinetic of Hsp90 plasma levels over time, following ALL injection. Data points represent mean of 3 animals. The cut-off Hsp90 value (0.1 ng/mL) is indicated. Asterisks represent starting week of sequential sacrifice of animals (Time Point 1). Some groups did not have enough animals for the experiment to be carried out until the third week of sacrifice. (C) Percentage of ALL cells (hCD45+) in bone marrow (BM) and peripheral blood (PB) at the different time points. Data points represent mean of 3 animals. Dotted line represents the usual cut-off (0.5%) for ALL detection by flow cytometry in blood samples. Different ALL cells are represented by colors. Circles; BCP-ALL. Triangles, T-ALL.

To address whether Hsp90 would be a useful marker for earlier detection of engraftment and to monitor the dynamic growth of leukemia in mice, groups of non-irradiated NOD/SCID mice injected with ALL were weekly monitored for plasma Hsp90 levels and sequentially sacrificed once the threshold of 0.1 ng/mL was reached. These animals were used to investigate leukemia cell proportion in bone marrow, liver, spleen and blood, in comparison to plasma Hsp90 levels. A scheme of this experiment is shown in Fig 2A.

As expected, Hsp90 levels in ALL-transplanted mice increased in a time dependent manner (Fig 2B). All three B-cell precursor ALL (two primary BCP-ALL and the RS4;11 cell line) reached Hsp90 levels above 0.1 ng/mL in the first week after transplantation. At first sight, T-cell ALL (two primary T-ALL and the TALL-1 cell line) had slower engraftment than BCP-ALL (Fig 2B). However, a closer examination showed that the percentage of T-ALL cells in bone marrow, liver, spleen and blood, at time point 1 (when median Hsp90 reached ≥ 0.1 ng/mL), tended to be equal or even higher in T-ALL than BCP-ALL (S1 Fig). Even though previously reported western blot analysis showed no difference in Hsp90 expression by BCP-ALL versus T-ALL [20], our results suggest that T-ALL may secrete/release lower amounts of Hsp90 into circulation than BCP-ALL, therefore giving false impression of a slower engraftment. In addition, levels of Hsp90 among different T-ALL were notably variable, thus absolute Hsp90 levels would not be reliable for comparisons among different T-ALL with respect to engraftment and progression in the NOD/SCID mouse.

Earlier studies have pointed to bone marrow and spleen as primary organs for ALL engraftment [2,23]. In agreement with a recent report showing primary BCP-ALL invasion and proliferation in the liver of non-irradiated NOG mice [7], our data indicate that liver is also one of the primary targets for ALL engraftment and progression in NOD/SCID mice (Fig 2C and S1 Fig).

At the first time point of sacrifice, median proportion of ALL cells in peripheral blood mononuclear cells was 0.01%, a percentage not detectable by flow cytometry unless mice were killed to obtain higher volume of blood. At the second time point (one week after time point 1), median ALL was 0.2% (Fig 2C), a percentage amenable to detection by flow cytometry even though being below the commonly used 0.5% cut-off value [3]. In the most conservative scenario, measuring plasma Hsp90 levels would allow at least one week anticipation on leukemia engraftment detection. At this earlier time of leukemia engraftment/progression, the percentage of ALL cells in BM was around 0.87%, but for some of the ALL samples, levels were below 0.1% (Fig 2C). Absolute number of ALL cells per animal (considering the sum of ALL cells in bone marrow, liver and spleen), in some cases was as low as 200 (S1 Fig, BCP-ALL#1 and TALL-1).

**Fig 3:**
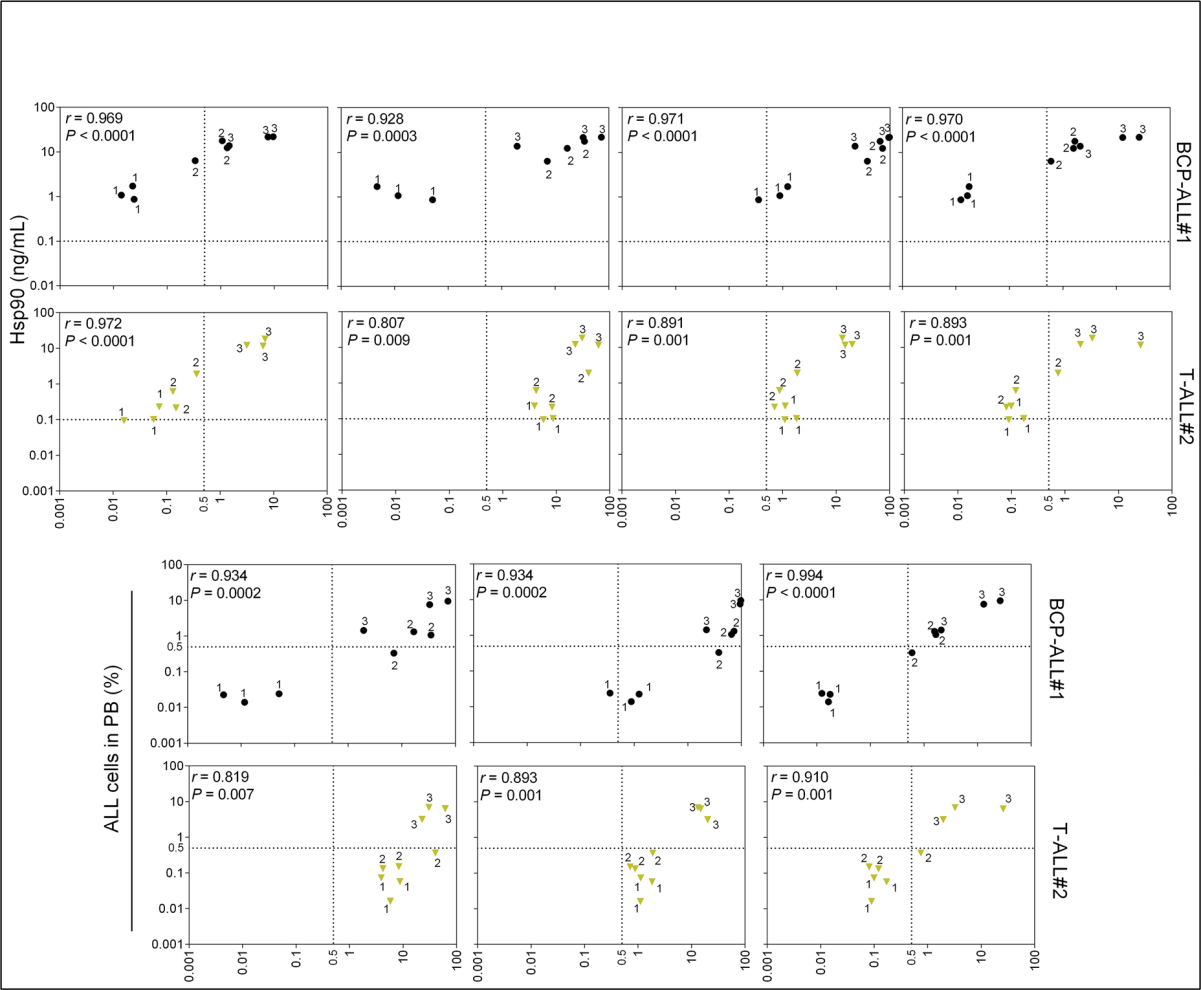
Correlation between plasma Hsp90 level and percentage of ALL cells in the different tissues analyzed. One representative case of three BCP-ALL or T-ALL analyzed is shown. For complete data refer to S2 Fig and S3 Fig ELISA Hsp90 and flow cytometry hCD45+ data were transformed to log10 and analyzed by Pearson’s correlation. Correlations between ALL in peripheral blood and in the different tissues are shown for comparisons. Dotted line represents cut-off values for ALL detection by flow cytometry (0.5%) or Hsp90 levels (0.1 ng/mL). Data points correspond to individual samples. Numbers near each data point represent time point of sampling (see Fig 2). PB; peripheral blood. BM; bone marrow. Circles, BCP-ALL. Triangles, T-ALL.

Notably, plasma Hsp90 levels were in strong linear correlation with percentage of BCP-ALL cells in the different tissues analyzed; even at very low leukemia burden (5 of the 6 evaluated groups of mice had blood ALL levels below 0.5%) (Fig 3A and S2 Fig). Contrary to our initial hypothesis, the percentage of ALL cells in peripheral blood (attainable by the analysis of large volume of blood) also strongly correlated with the percentage of ALL cells in other tissues analyzed, especially spleen (Fig 3B).

**Fig 4:**
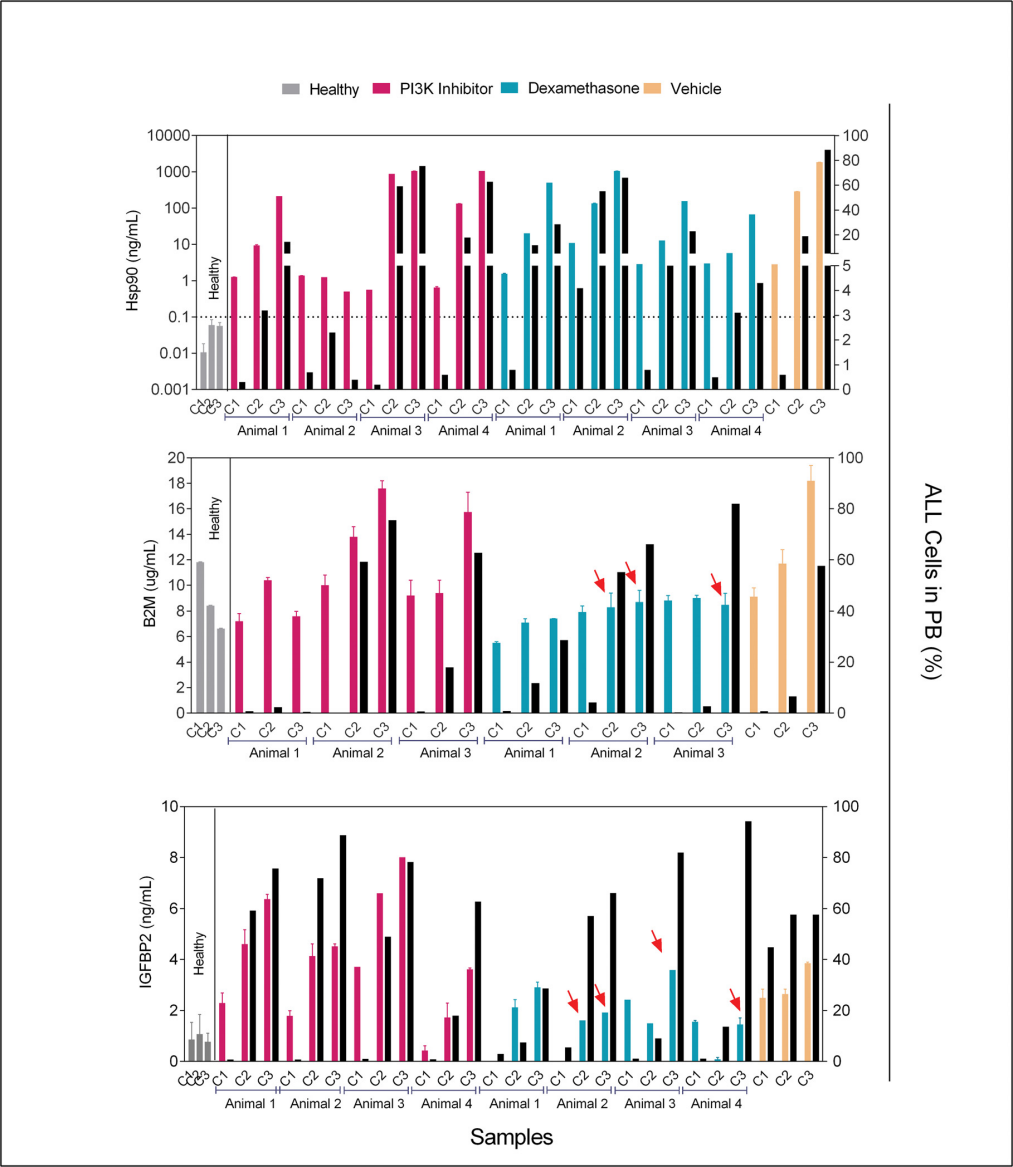
Chemotherapy effects on biomarkers levels. Plasma biomarkers levels (ELISA) and matched percentage of ALL cells (hCD45+) in peripheral blood (PB) from animals under chemotherapy treatment. Samples were collected for analysis at the beginning (C1 = day 5), middle (C2 = day 19) and end (C3 = day 33) of treatment. Colored bars represent mean ± SD of biomarker levels (left Y axis) for individual mice samples analyzed in duplicate. Different colors represent different treatments. The PI3K inhibitor used was AS605240. Black bars represent percentages of ALL cells in peripheral blood (right Y axis). The dotted line in the Hsp90 graphic represents the ELISA cut-off value. Red arrows highlight lower than expected ELISA values, when compared to levels found in untreated animals with similar percentage of ALL cells.

For T-ALL samples, 2 of 3 cells showed a clear linear correlation between Hsp90 levels and the percentage of ALL cells (Fig 3C and S3 Fig). Importantly, that particular T-ALL sample failed to show correlation between Hsp90 levels and leukemic load also failed in terms of percentage of ALL in peripheral blood and leukemic load. Moreover, data from three other primary T-ALL cases (one shown in Figs 1 and 4, and two shown in S4 Fig) showed good correlation between Hsp90 and leukemic load. In conclusion, although different T-ALL samples seemed to produce variable levels of Hsp90 in comparison to BCP-ALL that tended to be more homogenous, our data indicates that Hsp90 stands as a valid biomarker for both BCP- and T-ALL. Generally, these results validate the use of plasma Hsp90 levels to sequentially monitor engraftment and leukemia progression in NOD/SCID mice, at very earlier time points, when leukemia is at MRD levels [25] and analysis by flow cytometry would require the sacrifice of groups of animals at the different time points.

### Chemotherapy interference with *in vivo* production of Hsp90 by leukemia cells

If Hsp90 levels were used in preclinical evaluation of new anti-leukemia drugs or in mouse avatar assays, production of Hsp90 should parallel changes in leukemia cell numbers, with no direct influence of chemotherapy, i.e. chemotherapy should not interfere with transcription of the Hsp90 gene, translation of its mRNA, secretion of the Hsp90 protein, clearance of Hsp90 from serum, etc. To address this issue, animals were engrafted with a primary T-ALL and treated with dexamethasone, PI3K inhibitor (AS605240) or saline, after reaching ≥0.5% ALL in peripheral blood, considering that Hsp90 interacts directly with diverse glucocorticoids receptors [20,26] and PI3K inhibition significantly inhibits ALL metabolism [27]. The animals were treated every morning, Monday to Friday and on Friday afternoon; blood was collected for ELISA and flow cytometry analysis. Separate experiments were run for each of the three biomarkers. As shown in Fig 4, levels of Hsp90 were proportional to the percentage of ALL cells in peripheral blood, independently of treatment and B2M and IGFBP2 were not influenced by the PI3K inhibitor. Contrarily, production of both B2M and IGFBP2 by ALL cells in animals under dexamethasone treatment was lower than expected; indicating that decreases in circulating B2M and IGFBP2 levels following glucocorticoids treatment may not always reflect a corresponding reduction in leukemia cell numbers.

**Fig 5:**
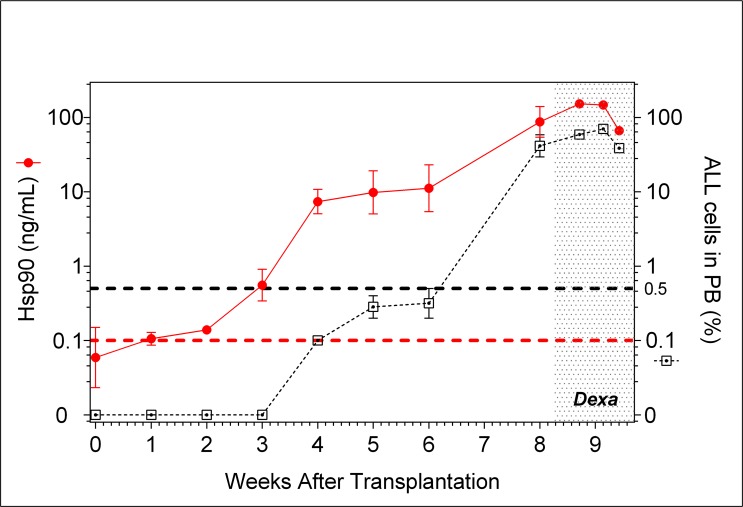
Kinetic of Hsp90 levels and percentage of peripheral blood ALL cells for BCP-ALL#3. Two mice were transplanted with primary BCP-ALL cells and assessed for peripheral blood hCD45+ cell percentage by flow cytometry and Hsp90 levels by ELISA. The animals were treated daily with dexamethasone after the seventh week of transplantation (dotted area). Red colored lines and symbols represent Hsp90 levels (left Y axis). Black lines and symbols represent the percentage of ALL cells in peripheral blood (right Y axis). There was no sampling in the seventh week. After the eighth week, peripheral blood samples were collected every three days.

This finding was confirmed with four other primary ALL (two BCP-ALL and two T-ALL). Engrafted animals were treated with dexamethasone after reaching ≥0.5% ALL in peripheral blood. Hsp90 levels were assessed and compared with percentage of ALL in peripheral blood during 3 weeks. For all ALL samples the correlation between Hsp90 levels and percentage of ALL cells in peripheral blood were highly significant (S4 Fig). Results for one of the BCP-ALL (#3) are shown in Fig 5, to illustrate how well Hsp90 levels correlate with the percentage of ALL cells in peripheral blood both before and during treatment of animals. Furthermore, it shows the usefulness of Hsp90 measurement for the earlier detection of ALL engraftment (second week versus sixth week by flow cytometry).

Although further experiments are needed to evaluate the effect of other chemotherapy drugs on in vivo production of Hsp90 by leukemia cells, Hsp90 is so highly abundant (1 to 2% of the cellular protein) that we are tempted to speculate that Hsp90 production would not be affected by chemotherapy. Indeed, even though Hsp90 is known to be upregulated in response to cellular stress, major differences in protein levels are at best 2–3 fold only [28].

In summary, we have validated Hsp90 as a soluble biomarker of ALL, for the earlier detection of leukemia engraftment and for monitoring leukemia kinetics, even at MRD levels and under chemotherapy treatment of mice.

## Supporting Information

S1 Fig Percentage and absolute numbers of ALL cells per tissue, at earlier time points of ALL progression. Groups of mice transplanted with ALL were weekly sacrificed to evaluate the preferred organ of leukemia engraftment and progression. Maximum volume of peripheral blood, as well as cells from bone marrow (femurs), liver and spleen were obtained. Post-ficoll mononuclear cells were analyzed as above. Total numbers of cells obtained were analyzed by flow cytometry for presence of ALL cells (hCD45+). For time points 1 and 2 see Fig 2. Bars represent mean values of 3 animals. PB; peripheral blood. BM; bone marrow.
(PDF)

S2 Fig Correlation between plasma Hsp90 level and percentage of BCP-ALL cells in the different tissues analyzed. ELISA Hsp90 and flow cytometry hCD45+ data from 3 different BCP-ALL were transformed to log10 and analyzed by Pearson’s correlation. Correlations between ALL in peripheral blood and in the different tissues are shown for comparisons. Dotted line represents the cut-off values for ALL detection by flow cytometry (0.5%) or Hsp90 levels (0.1 ng/mL). Data points correspond to individual samples. Numbers near each data point represent the time point of sampling (see Fig 2). PB; peripheral blood. BM; bone marrow.
(PDF)

S3 Fig Correlation between plasma Hsp90 level and percentage of T-ALL cells in the different tissues analyzed. ELISA Hsp90 and flow cytometry hCD45+ data from 3 different T-ALL were transformed to log10 and analyzed by Pearson’s correlation. Correlations between ALL in peripheral blood and in the different tissues are shown for comparisons. Dotted line represents cut-off values for ALL detection by flow cytometry (0.5%) or Hsp90 levels (0.1 ng/mL). Data points correspond to individual samples. Numbers near each data point represent the time point of sampling (see Fig 2). PB; peripheral blood. BM; bone marrow.
(PDF)

S4 Fig Correlation between plasma Hsp90 levels and percentage of ALL cells in the peripheral blood of animals before and during dexamethasone treatment. ELISA Hsp90 and flow cytometry hCD45+ data from animals transplanted with 2 different primary BCP-ALL or 2 different primary T-ALL. Data were transformed to log10 and analyzed by Pearson’s correlation. Data points correspond to individual samples. Black and red colors serve to differentiate among replicates (n = 2). Numbers near each data point represent the week after transplantation (for BCP-ALL#4 time points see Fig 5). Empty symbols represent samples collected before treatment initiation. Filled symbols represent samples collected under dexamethasone treatment. Dotted lines represent cut-off values for ALL detection by flow cytometry (0.5%) or Hsp90 levels (0.1 ng/mL). PB; peripheral blood.
(PDF)
