# Correction: Plasma Hsp90 Level as a Marker of Early Acute Lymphoblastic Leukemia Engraftment and Progression in Mice

**DOI:** 10.1371/journal.pone.0134774

**Published:** 2015-07-31

**Authors:** Mateus Milani, Angelo Brunelli Albertoni Laranjeira, Jaíra Ferreira de Vasconcellos, Silvia Regina Brandalise, Alexandre Eduardo Nowill, José Andrés Yunes

The X-axis labels are missing from Fig 3. The authors have provided a corrected version of Fig 3 here.

**Fig 3:**
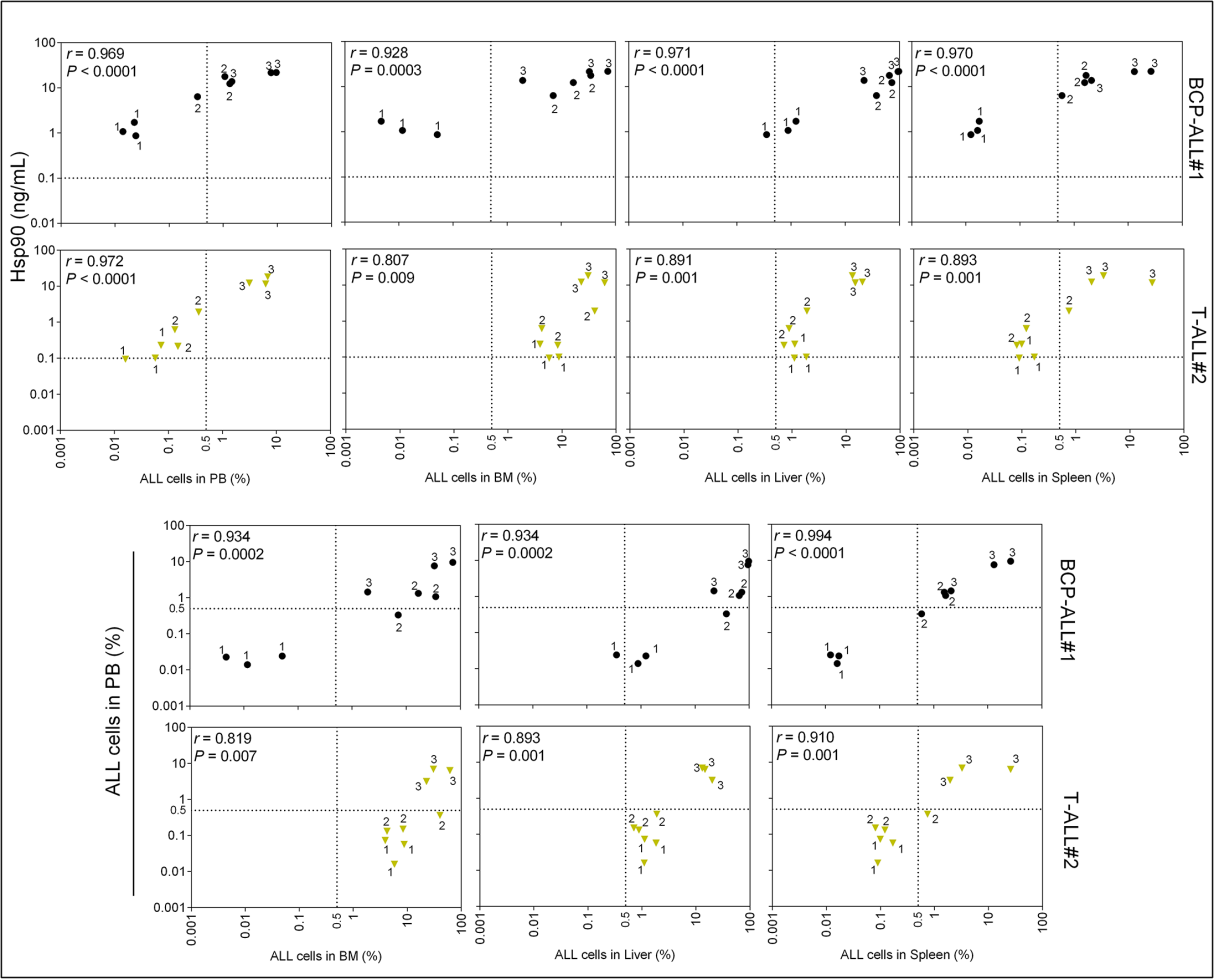
Correlation between plasma Hsp90 level and percentage of ALL cells in the different tissues analyzed. One representative case of three BCP-ALL or T-ALL analyzed is shown. For complete data refer to S2 Fig and S3 Fig ELISA Hsp90 and flow cytometry hCD45+ data were transformed to log10 and analyzed by Pearson’s correlation. Correlations between ALL in peripheral blood and in the different tissues are shown for comparisons. Dotted line represents cut-off values for ALL detection by flow cytometry (0.5%) or Hsp90 levels (0.1 ng/mL). Data points correspond to individual samples. Numbers near each data point represent time point of sampling (see Fig 2). PB; peripheral blood. BM; bone marrow. Circles, BCP-ALL. Triangles, T-ALL.
