# Correction: Plasma Hsp90 Level as a Marker of Early Acute Lymphoblastic Leukemia Engraftment and Progression in Mice

**DOI:** 10.1371/journal.pone.0138263

**Published:** 2015-09-11

**Authors:** The PLOS ONE Staff

## Notice of Republication

This article was republished on August 26, 2015, to correct the correction published on July 31, 2015. An incorrect version of Fig 3 was inadvertently used in the original correction. The publisher apologizes for the errors. Please download this article again to view the correct version. The originally published, uncorrected correction and the republished, corrected correction are provided here for reference.

## Supporting Information

S1 File Originally published, uncorrected article. (PDF)

S2 File Republished, corrected article. (PDF)
