# Supplementary figures and images for: Plasma Hsp90 Level as a Marker of Early Acute Lymphoblastic Leukemia Engraftment and Progression in Mice
Source: PLoS One. 2015 Jun 11;10(6):e0129298. doi: 10.1371/journal.pone.0129298 (PMC4466233; doi:10.1371/journal.pone.0129298)

Supplementary figure 1

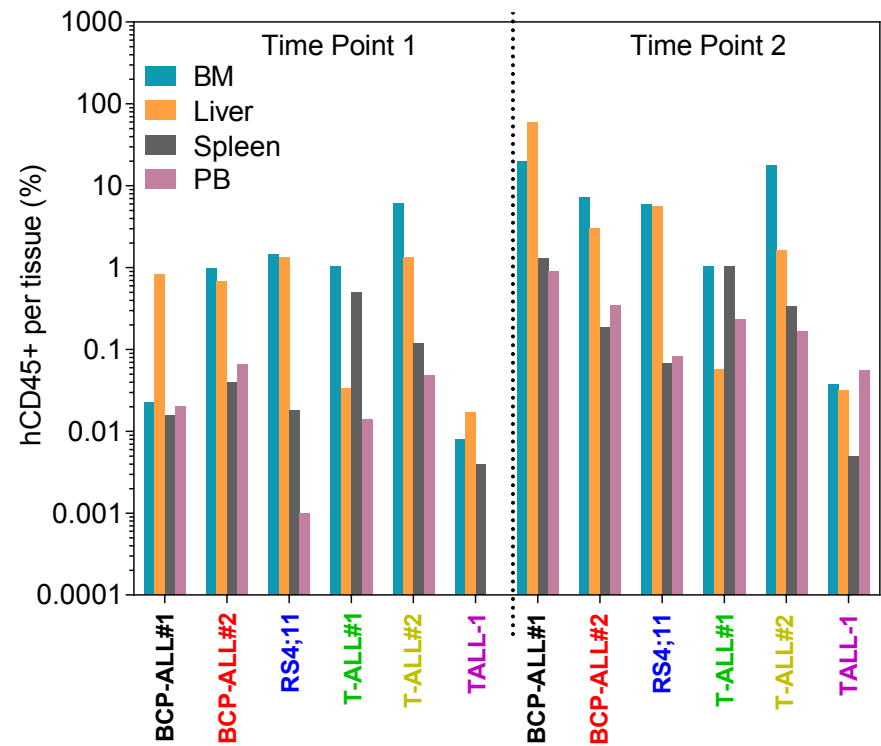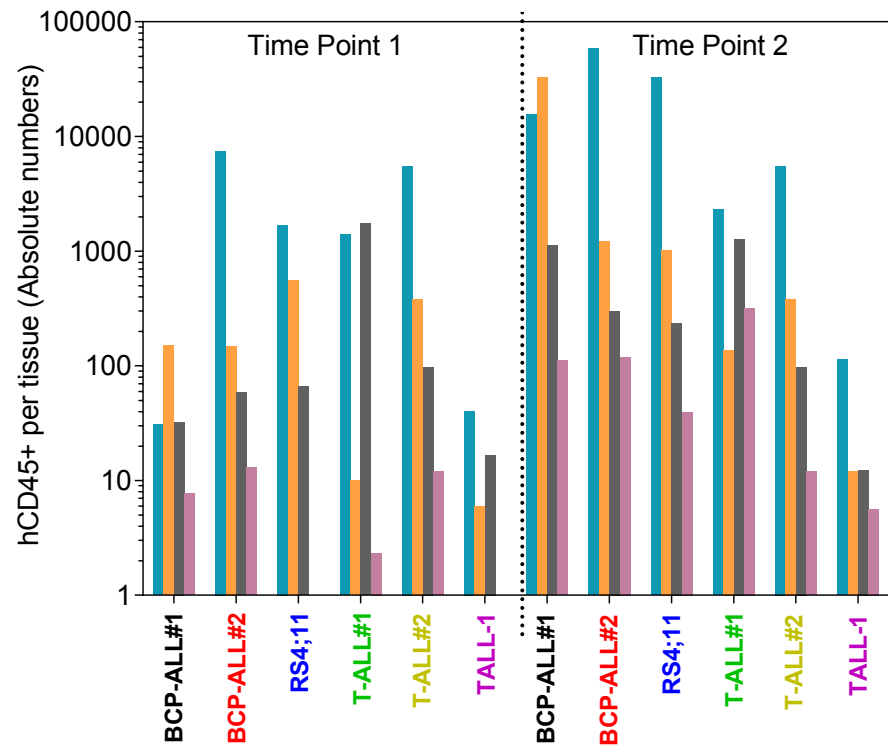

Supplement: S1 Fig — Groups of mice transplanted with ALL were weekly sacrificed to evaluate the preferred organ of leukemia engraftment and progression. Maximum volume of peripheral blood, as well as cells from bone marrow (femurs), liver and spleen were obtained. Post-ficoll mononuclear cells were analyzed as above. Total numbers of cells obtained were analyzed by flow cytometry for presence of ALL cells (hCD45+). For time points 1 and 2 see Fig 2. Bars represent mean values of 3 animals. PB; peripheral blood. BM; bone marrow. (PDF) [file pone.0129298.s001.pdf]

Supplementary figure 2

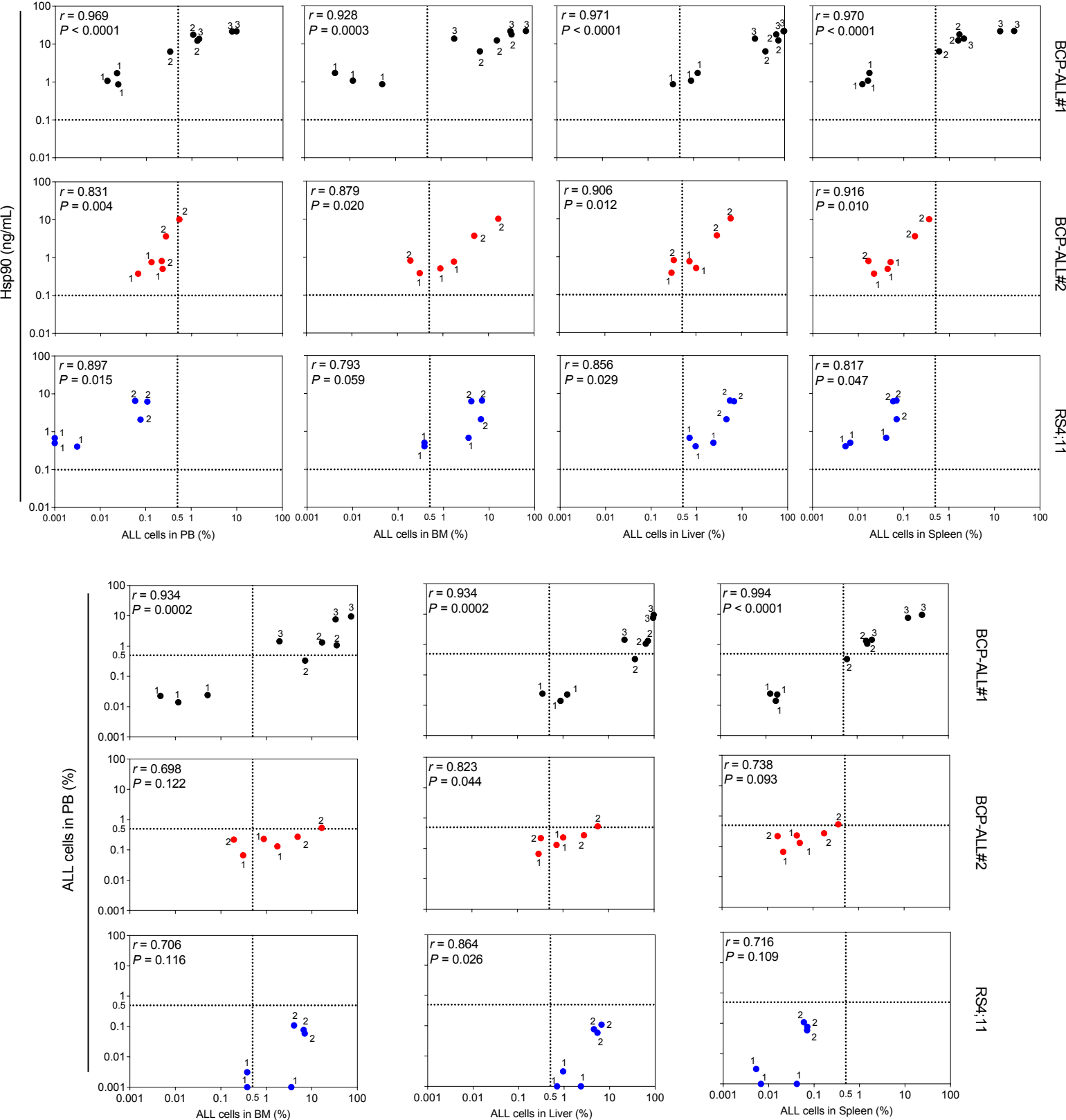

Supplement: S2 Fig — ELISA Hsp90 and flow cytometry hCD45+ data from 3 different BCP-ALL were transformed to log10 and analyzed by Pearson’s correlation. Correlations between ALL in peripheral blood and in the different tissues are shown for comparisons. Dotted line represents the cut-off values for ALL detection by flow cytometry (0.5%) or Hsp90 levels (0.1 ng/mL). Data points correspond to individual samples. Numbers near each data point represent the time point of sampling (see Fig 2). PB; peripheral blood. BM; bone marrow. (PDF) [file pone.0129298.s002.pdf]

Supplementary figure 3

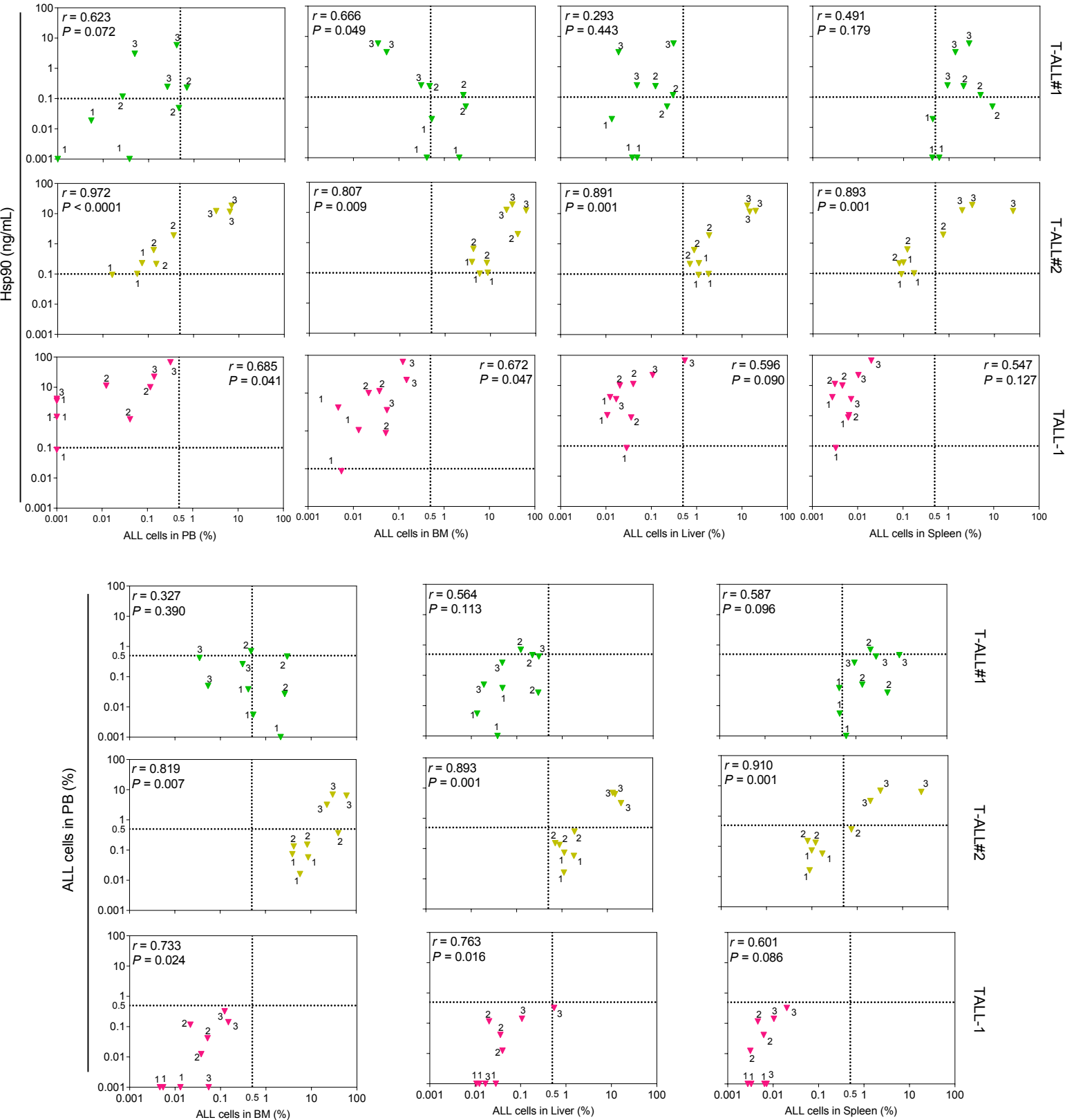

Supplement: S3 Fig — ELISA Hsp90 and flow cytometry hCD45+ data from 3 different T-ALL were transformed to log10 and analyzed by Pearson’s correlation. Correlations between ALL in peripheral blood and in the different tissues are shown for comparisons. Dotted line represents cut-off values for ALL detection by flow cytometry (0.5%) or Hsp90 levels (0.1 ng/mL). Data points correspond to individual samples. Numbers near each data point represent the time point of sampling (see Fig 2). PB; peripheral blood. BM; bone marrow. (PDF) [file pone.0129298.s003.pdf]

# Supplementary figure 4

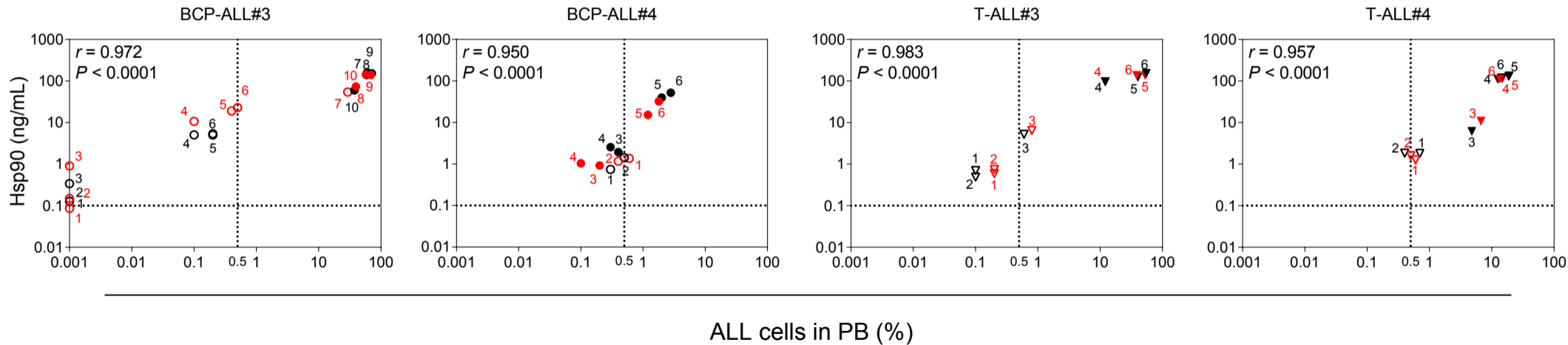

Supplement: S4 Fig — ELISA Hsp90 and flow cytometry hCD45+ data from animals transplanted with 2 different primary BCP-ALL or 2 different primary T-ALL. Data were transformed to log10 and analyzed by Pearson’s correlation. Data points correspond to individual samples. Black and red colors serve to differentiate among replicates (n = 2). Numbers near each data point represent the week after transplantation (for BCP-ALL#4 time points see Fig 5). Empty symbols represent samples collected before treatment initiation. Filled symbols represent samples collected under dexamethasone treatment. Dotted lines represent cut-off values for ALL detection by flow cytometry (0.5%) or Hsp90 levels (0.1 ng/mL). PB; peripheral blood. (PDF) [file pone.0129298.s004.pdf]
