# Supplementary material for: Correction: Plasma Hsp90 Level as a Marker of Early Acute Lymphoblastic Leukemia Engraftment and Progression in Mice
Source: PLoS One. 2015 Sep 11;10(9):e0138263. doi: 10.1371/journal.pone.0138263 (PMC4567283; doi:10.1371/journal.pone.0138263)
Supplement: S2 File — (PDF) [file pone.0138263.s002.pdf]

PLOS | ONE

CORRECTION

Correction: Plasma Hsp90 Level as a Marker of Early Acute Lymphoblastic Leukemia Engraftment and Progression in Mice

Mateus Milani, Angelo Brunelli Albertoni Laranjeira, Jaíra Ferreira de Vasconcellos, Silvia Regina Brandalise, Alexandre Eduardo Nowill, José Andrés Yunes

The X-axis labels are missing from Fig 3. The authors have provided a corrected version of Fig 3 here.

CrossMark
click for updates

OPEN ACCESS

Citation: Milani M, Laranjeira ABA, de Vasconcellos JF, Brandalise SR, Nowill AE, Yunes JA (2015) Correction: Plasma Hsp90 Level as a Marker of Early Acute Lymphoblastic Leukemia Engraftment and Progression in Mice. PLoS ONE 10(7): e0134774. doi:10.1371/journal.pone.0134774

Published: July 31, 2015

Copyright: © 2015 Milani et al. This is an open access article distributed under the terms of the Creative Commons Attribution License, which permits unrestricted use, distribution, and reproduction in any medium, provided the original author and source are credited.

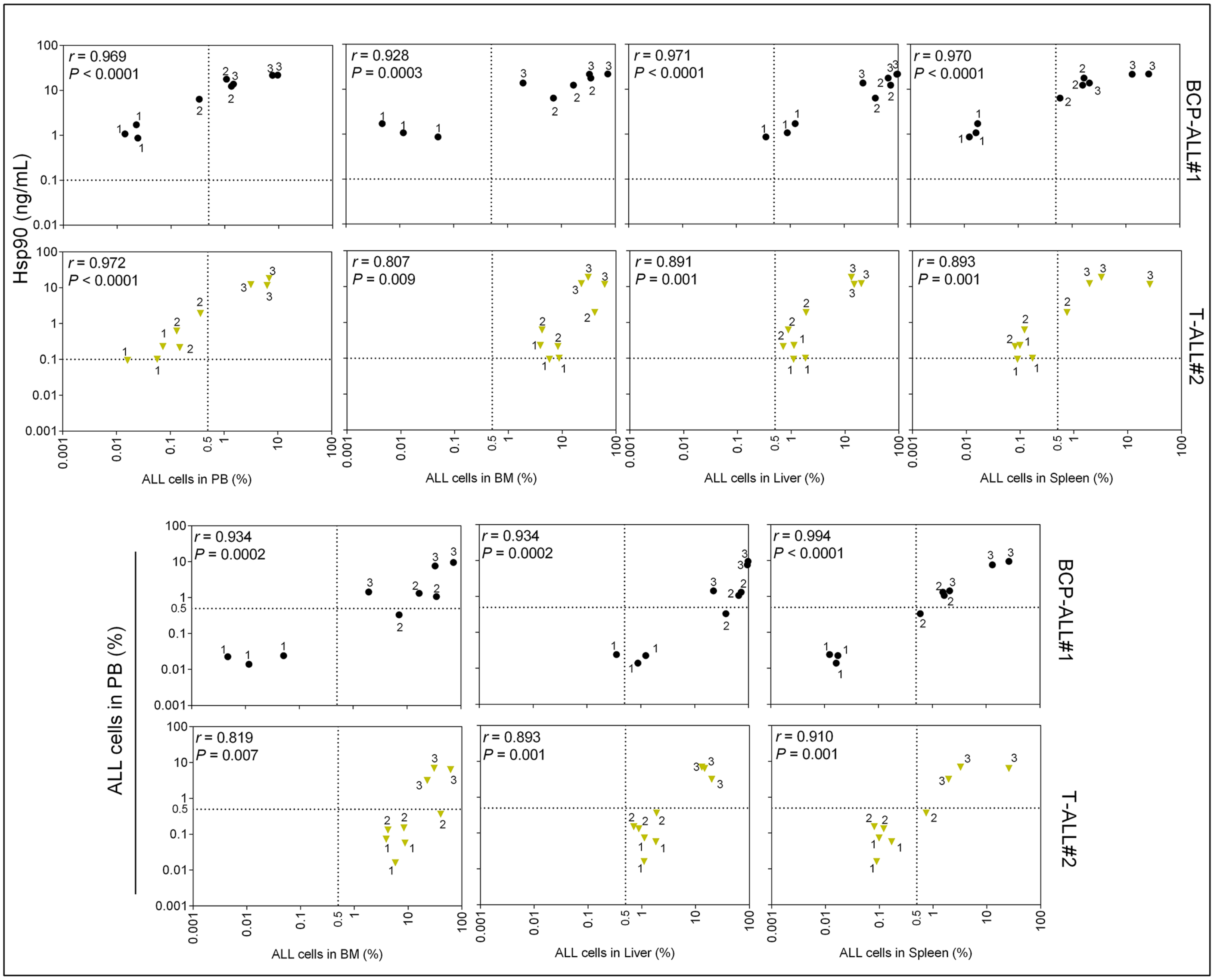

Fig 3. Correlation between plasma Hsp90 level and percentage of ALL cells in the different tissues analyzed. One representative case of three BCP-ALL or T-ALL analyzed is shown. For complete data refer to S2 Fig and S3 Fig ELISA Hsp90 and flow cytometry hCD45+ data were transformed to log10 and analyzed by Pearson's correlation. Correlations between ALL in peripheral blood and in the different tissues are shown for comparisons. Dotted line represents cut-off values for ALL detection by flow cytometry (0.5%) or Hsp90 levels (0.1 ng/mL). Data points correspond to individual samples. Numbers near each data point represent time point of sampling (see Fig 2). PB; peripheral blood. BM; bone marrow. Circles, BCP-ALL. Triangles, T-ALL.

doi:10.1371/journal.pone.0134774.g001

Reference

1. Milani M, Laranjeira ABA, de Vasconcellos JF, Brandalise SR, Nowill AE, Yunes JA (2015) Plasma Hsp90 Level as a Marker of Early Acute Lymphoblastic Leukemia Engraftment and Progression in Mice. PLoS ONE 10(6): e0129298. doi: 10.1371/journal.pone.0129298 PMID: 26068922
